# Improving the preanalytical stability of Alzheimer’s disease plasma biomarkers: the impact of blood collection tubes supplemented with protease inhibitors

**DOI:** 10.1002/alz.088796

**Published:** 2025-01-09

**Authors:** Yijun Chen, Xuemei Zeng, Jihui Lee, Anuradha Sehrawat, Tara K Lafferty, James J Boslett, William E Klunk, Tharick A. Pascoal, Victor L Villemagne, Ann D Cohen, Oscar L. Lopez, Nathan Yates, Thomas K Karikari

**Affiliations:** ^1^ University of Pittsburgh, Pittsburgh, PA USA; ^2^ University of Pittsburgh, PITTSBURGH, PA USA; ^3^ Department of Psychiatry, School of Medicine, University of Pittsburgh, Pittsburgh, PA USA

## Abstract

**Background:**

The detection and monitoring of Alzheimer's disease (AD) biomarkers in plasma are crucial for early diagnosis and prognosis. However, the stability of plasma AD biomarkers can be compromised by the degradation caused by endogenous proteases present in blood. The efficacy of protease inhibitors in mitigating this degradation is yet to be established. This study evaluated the stability of five plasma AD biomarkers (GFAP, NFL, Aβ1‐40, Aβ1‐42, and pTau181) in plasma over time under room temperature (RT) conditions. Samples were collected concurrently into either standard K2EDTA or BD‐P100 tubes, with the latter being coated with protease inhibitors. Our objective was to assess the effectiveness of protease inhibitors in preserving the stability of plasma AD biomarkers in plasma.

**Method:**

We evaluated two experimental approaches. In Approach 1, whole blood samples (n=16) from the same draw were collected into K2EDTA and BD‐P100 tubes and stored at RT for 0h and 24h. In Approach 2, plasma samples (n=19) were isolated from whole blood collected into BD‐P100 and K2EDTA tubes and immediately stored at RT for 0h, and 24h. The levels of plasma AD biomarkers were measured using immunoprecipitation with mass spectrometry (IP‐MS) and Single Molecule Array (SIMOA). The IP‐MS assay replicated a protocol (Nakamura et al., 2018) for Aβ1‐40 and Aβ1‐42 measurement.

**Result:**

The IP‐MS assay indicated that the use of BD‐P100 tubes significantly reduced Aβ peptide degradation compared with the K2EDTA tube. In Approach 1, the IP‐MS Aβ signal decreased by 23‐34% for samples collected in K2EDTA tubes relative to 17‐18% for samples collected in BD‐P100 tubes. In Approach 2, the IP‐MS Aβ peptide signal decreased by 14‐16% for K2EDTA tubes, with a lower decrease of 3‐7% for BD‐P100 tubes. The SIMOA assays produced similar results for Aβ peptides. No significant variations were detected in time‐dependent pTau181, GFAP, and NFL levels for samples collected using both tubes.

**Conclusion:**

The study showed that tubes supplemented with protease inhibitors significantly improve the preanalytical stability of Aβ1‐40 and Aβ1‐42 in plasma. These results have important implications for preanalytical procedures that are needed to enable widespread access and utilization of plasma biomarkers in resource‐limited settings.

